# Characterization of cyclic alternating pattern in infants with laryngomalacia

**DOI:** 10.5935/1984-0063.20210017

**Published:** 2022

**Authors:** Laura Mendoza Cáceres, Ángel Daniel Santana Vargas, Gabriela Millán Rosas, Eduardo Barragán Pérez, Adrián Poblano, Rafael Santana Miranda

**Affiliations:** 1 Universidad Nacional Autónoma de México, Facultad de Medicina, Sleep disorders Clinic - Mexico City - Mexico.; 2 General Hospital of Mexico, Department of Research - Mexico City - Mexico.; 3 Hospital Infantil de México Federico Gómez, Pediatric Neurology Department - Mexico City - Mexico.; 4 National Institute of Rehabilitation, Laboratory of Cognitive Neurophysiology - Mexico City - Mexico.s

**Keywords:** Laryngomalacia, Sleep, Apnea, Infant, Newborn, Diseases

## Abstract

**Objective:**

Cyclic alternanting pattern (CAP) has been considered a marker of sleep instability in children. The aim of this study was to evaluate the CAP in infants with laryngomalacia.

**Material and Methods:**

CAP were quantified in 15 infants with laryngomalacia (mean age 167.2±97.21 days) and 10 controls (mean age of 158.5±116.2 days) using polysomnography.

**Results:**

The distribution of the A2 subtypes across NREM stages in infants with laryngomalacia showed a decrease, as well as in the mean duration of CAP sequences. The A3 CAP and arousals increased in infants with laryngomalacia. Our data showed a stronger correlation between the mean duration of A1 CAP and the age in healthy controls than in infants with laryngomalacia. In accordance to previous reports infants with laryngomalacia exhibited an increase in total awake time, apnea-hypopnea index, and a decrease in N3 stage compared to controls.

**Discussion:**

Our findings add to a growing body of literature of CAP as an indicator of brain maturation

## INTRODUCTION

Cyclic alternating pattern (CAP) is a normal rhythm present in electroencephalography (EEG) recordings during non-rapid eye movement (NREM) sleep commonly observed in healthy subjects. CAP is characterized by periodic bursts of transient EEG activation phases (phase A) followed by inhibition phases (phase B)^1^. CAP has been related with brain maturation and several studies observed alterations in CAP pattern in children with neurodevelopmental diseases such as congenital hypothyroidism, Prader Willi Syndrome, Asperger syndrome, Down syndrome, Rolandic, and Dravet epilepsy^2,3,4,5,6^. Under these conditions, the lower percentage of the A1 phase and an increase of the A2 phase is considered a refection of higher NREM sleep instability and an indirect indicator of poor brain maturation^7,8,9^.

Laryngomalacia is the most common cause of infant stridor that can be a consequence of an underdeveloped or abnormally integrated central nervous system (CNS)^10,11^. According to this idea, sleep development is an essential indicator of the early development of the human CNS. Deepness of sleep is related to the maturation of slow-wave activity^7^, which occurs paralleled by massive synaptic remodeling and cortical maturation^8^, and a progressive increase in the CAP rate^9^. In addition, obstructive sleep apnea (OSA) is sometimes found concurrently in patients with laryngomalacia further affecting the brain development^10^.

It is widely accepted that CAP is an important factor which correlates with neurophysiological aspects of sleep and cognition in children^11,12,13^. Sleep parameters has been studied in infants with laryngomalacia before and after supraglottoplasty. In infants with severe laryngomalacia it is reported a complete polysomnographic study prior to and after supraglottoplasty, in which authors described that surgery reduced the apnoea-hypoapnoea index (AHI), as well as an increase of sleep efficiency^14^. However, nothings is known about the CAP in infants with laryngomalacia and the analysis of sleep microstructure could provide important data regarding the instability, sleep fragmentation, maturation, and EEG development. Thus, the aim of our work was to provide a full evaluation of macro and microstructure of sleep in infants with laryngomalacia in comparison to healthy controls.

## MATERIAL AND METHODS

### Participants

This study took place at the Sleep Disorder Clinic at the Universidad Nacional Autónoma de México. For this study, 15 infants from 1 to 12 months of age (females 53%, mean age of 158.5±116.2 days) with laryngomalacia confirmed by an otolaryngologist carried out by Flexible Laryngoscopy at the Hospital Infantil de México Federico Gómez and with no previous surgical treatment or other diseases were subjected to polysomnographic recording. The control group consisted of 10 low risk infants (females 40%, the mean age of 167.2±97.21 days) with no laryngomalacia in order to follow the neurodevelopment during the first 2 years.

Exclusion criteria were: no surgical intervention or other chronic diseases. The research and ethics committees of the participant’s institutions approved the study, and parents signed an informed consent letter after a full explanation of the objectives of the study following recommendations of the declaration of Helsinki.

### Polysomnographic study

The polysomnographic recordings were performed at the clinic of sleep disorders. All infants were scheduled for a study in the morning. Silverplate electrodes for electroencephalographic (EEG) recording were placed in scalp following the International 10-20 System at F1, C3, T3, O1, F2, C4, T4, O2, and Cz sites. Other electrodes were used to study right and left eye movement, electromyography (EMG) at the chin muscle, oronasal thermal airfow, and thoracic and abdominal belts for respiratory activity. Infants were allowed to sleep for recordings of at least 2 hours. This procedure allowed us to record at least two sleep cycles. Polysomnographic recordings in all infants started between 08:00 and 08:30 and usually lasted until 10:30 and 11:00 hours. The scoring of each sleep stage was blindly done following the international guidelines used for infants and children^15,16^. Sleep architecture was classified into three NREM sleep stages (N, N1, N2, and N3) and REM sleep. The following variables were studied: total sleep recording, total wake time, total sleep time, total time in REM sleep, total time in NREM sleep, total time in N1+ N2 sleep, and total time in N3 sleep. We also evaluated the apnea/hypopnea index, oxygen saturation, and arousal index.

### Cyclic alternating pattern (CAP) scoring

The CAP is defined as a rhythm present in NREM sleep characterized by EEG activity with sequences of transient electro-cortical activations (phase A of the cycle) different from EEG background activity (phase B of the cycle). These sequences are repeated several times during the night in a cyclic pattern interrupted by stable sleep without oscillations, called non-CAP phase longer than 60 seconds. The A phases of the CAP were subdivided into different subtypes: A1, A2, and A3, based on their frequency content. Subtype A1 was composed predominantly by slow waves (EEG synchrony), subtype A3 with the prevalence of fast EEG activities (EEG desynchrony), and subtype A2 presenting a combination of both^7^. CAP scoring was manually blindly performed by two qualified neurophysiologists, based on the Atlas of qualification of Terzano^16^, CAP parameters studied were as follows: CAP rate, CAP time, index of each CAP subtype, percentage of each subtype, mean duration of each subtype, and CAP sequences.

### Statistical analysis

Statistical analyses were carried out using the package IBM SPSS Statistics for Windows version 19 (Armonk, NY: IBM Corp.) Mean and the standard deviation was calculated for quantitative variables; meanwhile, frequencies and percentages were calculated for qualitative variables. Levene’s test evaluated the homoscedasticity of the distribution in each variable. “U” of Mann-Whitney test was used to compare means between groups. For multiple comparisons, Bonferroni corrections were used to avoid infation of calculations. Linear simple correlation analysis among age and CAP parameters was calculated. A *p*≤0.05 was used to accept differences as significant.

## RESULTS

### Laryngomalacia is associated with changes in sleep architecture

The main findings showed that infants with laryngomalacia presented longer total awake time (*p*=0.005) and shorter sleep in the N3 stage (*p*=0.041) when compared to controls. In addition, infants with laryngomalacia presented an increased number of arousals (*p*<0.01). Apnea/hypopnea index, hypopnea index, and mixed apnea index showed increased scores in infants with laryngomalacia (*p*=0.001, *p*<0.001, and *p*=0.004, respectively), meanwhile the mean of oxygen saturation was higher in infants with laryngomalacia than controls (*p*=0.014); however, no differences were found in REM, NREM light stages, and other parameters of sleep macrostructure (Table 1).

**Table 1:** Polysomnography, arousal and respiratory data from control and patients with laryngomalacia Abbreviations. SD = standard deviation. AHI = apnea/hypopnea index. OAI = obstructive apnea index. HI hypopnea index. MAI = mixed apnea index. CAI = central apnea index. SO2 = saturation of oxygen. Bold characters indicate statistically significant.

	Laryngomalacia n = 15			Control n = 10			p
	Mean	SD	Median	Mean	SD	Median	
Age (days)	158.80	116.20	180.00	167.20	97.21	165.00	.723
Total recording							
time (min)	129.93	11.07	129.00	114.12	24.51	119.55	.091
**Total awake time**	**28.70**	**20.26**	**21.50**	**9.65**	**7.89**	**10.00**	**.005**
Total sleep time	101.23	20.40	106.00	101.50	24.95	113.50	.495
Total sleep time in							
REM (min)	26.80	13.19	25.50	23.50	8.71	22.75	.461
Total sleep time							
nREM (min)	74.13	19.97	71.50	79.00	24.80	91.25	.285
Total sleep time in							
N1 + N2 stages	15.03	15.81	17.50	3.85	3.33	4.00	.397
**Total sleep time in N3 stage (min)**	**55.97**	**17.01**	**57.00**	**75.15**	**25.55**	**86.25**	**.041**
Arousal index (no./h)	16.63	7.59	14.60	6.86	2.25	6.48	.000
**AHI (no./h)**	**24.70**	**21.21**	**24.60**	**5.22**	**1.50**	**5.70**	**.001**
OAI (no./h)	2.15	3.52	0.00	0.14	0.44	0.00	.160
**HI (no/h)**	**16.19**	**15.55**	**10.50**	**0.48**	**1.16**	**0.00**	**.000**
**MAI (no/h)**	**1.17**	**1.51**	**0.40**	**0.00**	**0.00**	**0.00**	**.004**
**CAI (no/h)**							
2.79	2.91	1.50	4.61	1.36	4.80	.023	
SO^2^ Mean(%)	93.61	2.78	94.50	90.77	2.49	90.98	.014SO2
respiratory/event	88.2700	4.83000	90.0000	88.2100	2.57000	88.5000	0.6820
SO2 Minimal respiratory event	80.7300	10.85000	82.0000	86.4400	2.71000	87.0000	0.1510

### The cyclic alternating pattern is altered in infants with laryngomalacia

Infants with laryngomalacia showed a lower CAP A2 percentage and A2 index (*p*<0.001 and *p*=0.001, respectively) in comparison to controls. Isolated phase A and mean CAP sequence also showed lower scores in infants with laryngomalacia. No difference was found in CAP rate in infants with laryngomalacia, compared with the control group as well as other parameters of CAP (Table 2).

**Table 2:** Cyclic alternating pattern (CAP) parameters from laryngomalacia and control groups. Abbreviations. SD = standard deviation. CAP = cyclic alternation pattern. Bold characters indicate statistically significant.

	Laryngomalacia n = 15			Control n = 10			p
	Mean	SD	Median	Mean	SD	Median	
CAP rate %	53.96	15.94	55.38	58.02	17.40	54.52	.683
Total CAP time (min)	40.19	16.05	39.72	43.61	14.94	46.54	.683
**A2**%	**2.54**	**1.71**	**2.36**	**7.81**	**3.76**	**7.52**	**.000**
A1 index (number/h)	69.75	21.13	72.86	64.64	21.32	21.32	.807
A3 index (number/h)	16.02	8.15	13.26	13.38	6.54	12.10	.397
**Mean duration CAP B (s)**	**19.86**	**5.42**	**18.61**	**23.06**	**3.87**	**21.36**	**.048**
Mean duration CAP A2 (s)	8.14	6.09	7.33	6.26	1.34	6.06	.605
**Isolated A phase (number)**	**3.33**	**2.13**	**3.00**	**16.90**	**7.34**	**17.00**	**.000**

### Correlation between A1 type and age is altered in infants with laryngomalacia

**Figure 1:**
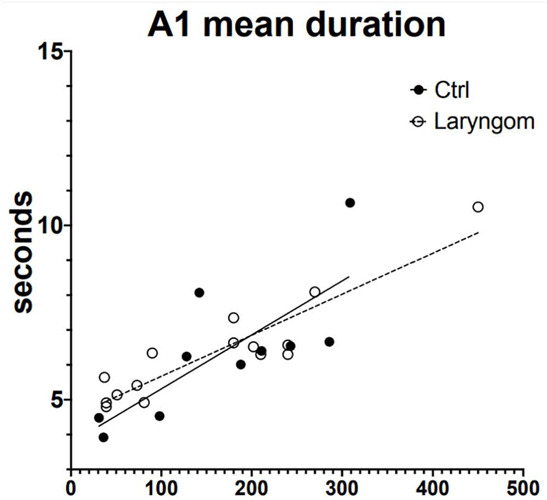
Linear simple regression calculation between Cyclic alternating pattern (CAP) A1 mean duration (up) and CAP rate (down) with age in infants with laryngomalacia (open cicles) and controls (black circles), and regression lines (discontinuous line = infants with laryngomalacia; continuous line = controls). Equations calculations showed a stronger score for control infants.

A significant positive correlation between A1 mean duration and age was found in infants with laryngomalacia and controls (*p*<0.001 in both groups). In infants with laryngomalacia, correlations was stronger than in controls (R^2^=0.8433 and R^2^=0.592, respectively) and CAP rate (laryngomalacia R^2^=0.715, *p*=0.002 vs. control R^2^=0.554, *p*=0.001) (Figure 1). No significant correlation was found in other CAP parameters.

## DISCUSSION

Lar yngomalacia is the most common congenital laryngeal abnor mality and may be associated with OSA, sleep disturbances, and possibly cognitive and behavioral disturbances. Infants with severe laryngomalacia present an apnea-hypopnea index within the range of severe OSA, which can be addressed after supraglottoplasty^14,18^. We reported here for the first time that sleep microarchitecture is altered in infants with laryngomalacia and our results corroborate that infants with laryngomalacia exhibit a higher apnea/hypopnea index, hypopnea index, mixed apnea index, central apnea index, and alterations in sleep architecture that could be generated indirectly by arousability and lower slow-wave sleep (N3)^2^.

Similar results were found in infants with congenital hypothyroidism, which exhibited a higher frequency of central apnea, hypopnea, and arousals. Those patients had a higher frequency in the percentage of A3 subtype and arousals with electroencephalographic desynchrony. However, these patients presented a lower percentage of A1 subtype. Moreover, infants with congenital hypothyroidism showed a positive correlation between A1 mean duration and age, which was stronger in the control group than in the congenital hypothyroidism group^2^. Infants with congenital hypothyroidism also showed a smaller slope when compared to healthy controls.

Despite the previous observations in patients with congenital hypothyroidism, the respiratory centers at brainstem level (central apneas) and the thalamocortical activity (number and average duration of A1 are compromised^2^; meanwhile, in infants with laryngomalacia breathing after birth is compromised and the predominant respiratory events have a partial obstructive component (hypopneas), central and mixed components affecting cortical development (duration mean of CAP subtype A1 concerning age). In this way, the stronger correlation between A1 mean duration and age as well as the higher slope in laryngomalacia group in A1 index, which could be interpretate as an early predictor of poor brain development in case the surgery do not be executed as soon as possible after diagnosis.

According to the previous observations we propose that sleep fragmentation in infants with laryngomalacia is presented as a clear higher arousability secondary to sleep disorder breathing with predominance to partial obstructive events meanwhile the ontogenetic role of CAP could be disrupted by a higher mean duration of CAP sequences, difference in the positive correlation between A1 mean duration and age, lower scores of CAP A2, isolated phases A, and marginal significance higher CAP phase A3 associated to the arousability in infants.

It is known that polysomnographic studies in infants with laryngomalacia are evaluated mainly before and after surgical procedures. However, since sleep has an important ontogenetic role in neuro-development^19^, clinicians must be alert for developmental and cognitive milestones altered in infants with laryngomalacia, including the evaluation of CAP as an early indicator of neurodevelopmental handicaps, to have an early opportunity to treat infants with laryngomalacia and decrease the risk of neurodevelopment alterations.

Despite the small sample size, we were able to study the sleep-microstructure in infants with laryngomalacia and found relevant information for future analysis.
